# Continuous lenalidomide and low‐dose dexamethasone in patients with transplant‐ineligible newly diagnosed MM: FIRST trial subanalysis of Canadian/US patients

**DOI:** 10.1002/cam4.3511

**Published:** 2020-10-13

**Authors:** Andrew Belch, Nizar Bahlis, Darrell White, Matthew Cheung, Christine Chen, Chaim Shustik, Kevin Song, Axel Tosikyan, Angela Dispenzieri, Kenneth Anderson, Diane Brown, Suzanne Robinson, Shankar Srinivasan, Thierry Facon

**Affiliations:** ^1^ Cross Cancer Institute Edmonton AB Canada; ^2^ Tom Baker Cancer Centre Calgary AB Canada; ^3^ Dalhousie University and Queen Elizabeth II Health Sciences Centre Halifax NS Canada; ^4^ Odette Cancer Centre Toronto ON Canada; ^5^ Princess Margaret Cancer Centre Toronto ON Canada; ^6^ McGill University Health Centre Montréal QC Canada; ^7^ Leukemia/Bone Marrow Transplant Program of British Columbia Division of Hematology Vancouver General Hospital Vancouver BC Canada; ^8^ Hôpital du Sacré‐Coeur de Montréal Montréal QC Canada; ^9^ Mayo Clinic Rochester MN USA; ^10^ Dana‐Farber Cancer Institute Boston MA USA; ^11^ Celgene, a Bristol‐Myers Squibb Company Mississauga ON Canada; ^12^ Celgene, a Bristol‐Myers Squibb Company Boudry Switzerland; ^13^ Bristol Myers Squibb Princeton NJ USA; ^14^ Service des Maladies du Sang Hôpital Claude Huriez Lille France

**Keywords:** Canada, lenalidomide, newly diagnosed multiple myeloma, transplant‐ineligible, United States

## Abstract

The phase 3 FIRST trial demonstrated significant improvement in progression‐free survival (PFS) and overall survival (OS) with an immune‐stimulatory agent, lenalidomide, in combination with low‐dose dexamethasone until disease progression (Rd continuous) vs melphalan +prednisone + thalidomide (MPT) in transplant‐ineligible patients with newly diagnosed multiple myeloma (NDMM). Rd continuous similarly extended PFS vs fixed‐duration Rd for 18 cycles (Rd18). Outcomes in the Canadian/US subgroup (104 patients per arm) are reported in this analysis. Rd continuous demonstrated a significant improvement in PFS vs MPT (median, 29.3 vs 20.2 months; HR, 0.69 [95% CI, 0.49‐0.97]; *p* = 0.03326) and an improvement vs Rd18 (median, 21.9 months). Median OS was 56.9 vs 46.8 months with Rd continuous vs MPT (*p* = 0.15346) and 59.5 months with Rd18. The overall response rate was higher with Rd continuous and Rd18 (78.8% and 79.8%) vs MPT (65.4%). In the 49.0%, 52.9%, and 29.8% of patients with at least very good partial response in the Rd continuous, Rd18, and MPT arms, respectively, the median PFS was 56.0, 30.9, and 40.2 months, respectively. The most common grade 3/4 treatment‐emergent adverse events were neutropenia (28.4%, 30.1%, and 52.0%), anemia (23.5%, 21.4%, and 23.5%), and infections (37.3%, 30.1%, and 24.5%) with Rd continuous, Rd18, and MPT, respectively. These results were consistent with those in the intent‐to‐treat population, confirming the benefit of Rd continuous vs MPT in the Canadian/US subgroup and supporting the role of Rd continuous as a standard of care for transplant‐ineligible patients with NDMM.

## Graphical abstract

In this Canadian/US subgroup analysis of the FIRST trial, lenalidomide +low‐dose dexamethasone until disease progression (Rd continuous) extended median PFS by 9 months, achieved deeper responses (≈20% increase in ≥VGPR rate), and delayed median TTNT by >14 months vs melphalan +prednisone + thalidomide (MPT). Safety results were generally consistent with the intent‐to‐treat analysis. The results support the role of Rd continuous as a standard of care for transplant‐ineligible patients with NDMM.

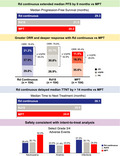

## 1 INTRODUCTION

Multiple myeloma (MM) is a plasma cell neoplasm characterized by hyperproliferation of malignant plasma cells in the bone marrow and immune dysfunction, which reduces the patient's ability to fight the disease.^1^, ^2^, ^3^, ^4^ However, an improved understanding of the immune microenvironment and role of the host immune system is informing treatment strategies and has led to reconsideration of cytotoxic therapies, like melphalan, due to their negative impact on immune function.^5^, ^6^

Agents with immune‐stimulatory effects, notably lenalidomide and pomalidomide, have been explored in the MM setting. These agents exhibit multifaceted antimyeloma activity by enhancing immune function, exhibiting direct antitumor (i.e., tumoricidal) effects, and disrupting aberrant stromal cell support by decreasing cytokine production from interactions between stromal and MM cells.^7^ Specifically, lenalidomide has been shown to modulate the immune response, in part by increasing the activity and number of T and natural killer cells.^8^, ^9^, ^10^ These features support the role of immunomodulatory treatment early in the course of disease to help improve the immune system and may explain the observed improvement in patient outcomes.

Lenalidomide +dexamethasone (Rd) has been a mainstay of treatment in the newly diagnosed MM (NDMM) setting for a number of years. The FIRST study (N = 1623) evaluated lenalidomide +dexamethasone (Rd) in transplant‐ineligible NDMM across 18 countries in Europe, North America, and the Asia‐Pacific region.^11^, ^12^ Patients received Rd until disease progression (Rd continuous), Rd for 18 cycles (Rd18), or melphalan +prednisone + thalidomide (MPT) (Rd18 and MPT both had a duration of 72 weeks). At the final analysis, median progression‐free survival (PFS) was significantly higher with Rd continuous vs MPT (26.0 vs 21.9 months; *p* < 0.00001), with a 21.0‐month median PFS for Rd18. Furthermore, Rd continuous delayed time to next antimyeloma treatment (TTNT) vs Rd18 and MPT (median, 36.7 vs 28.5 and 26.7 months, respectively). The median overall survival (OS) was also significantly higher with Rd continuous vs MPT (59.1 vs 49.1 months; *p* = 0.0023), and the median OS with Rd18 was 62.3 months. The overall response rate (ORR) was higher with Rd continuous (81%) vs MPT (67%) and similar to that with Rd18 (79%).

The inclusion of the Rd18 arm in FIRST enabled evaluation of continuous vs fixed‐duration treatment.^12^ In addition to prolonging PFS in the intent‐to‐treat (ITT) population, Rd continuous also achieved significantly more durable responses and prolonged PFS in each response subgroup (including those with complete response [CR]) vs MPT or Rd18.^13^ The benefit of continuous treatment has also been demonstrated in a pooled analysis of three phase 3 NDMM trials, in which it significantly improved survival outcomes vs fixed‐duration treatment.^14^

The above results support the use of frontline continuous oral therapy for transplant‐ineligible patients with NDMM and established Rd continuous as a standard of care, as reflected by clinical guidelines in Canada, the United States, and Europe.^15^, ^16^, ^17^ Furthermore, Rd is now commonly used in combination with other agents in many triplet regimens.

Treatment patterns can vary due to multiple factors, including geography and local guidelines. Thus, this analysis evaluated the outcomes in the subgroup of FIRST patients from Canada and the United States.

## 2 METHODS

### 2.1 Study design

Details of the FIRST study have been published previously.^11^, ^12^ Briefly, patients in the global phase 3 study were stratified by age (≤75 vs >75 years), International Staging System disease stage (I/II vs III), and country, and then randomized 1:1:1 to receive open‐label Rd continuous, Rd18, or MPT. Key inclusion criteria included age ≥18 years, Eastern Cooperative Oncology Group performance status (ECOG PS) ≤2, and no prior treatment for symptomatic and measurable transplant‐ineligible NDMM.

The primary endpoint was PFS, and the primary comparison was between the Rd continuous and MPT arms (PFS comparison of Rd continuous vs Rd18 arms and MPT vs Rd18 arms was a secondary objective). OS was the key secondary endpoint. ORR, TTNT (which censors deaths), and safety, including second primary malignancies (SPMs), were also secondary endpoints. Time from randomization to second progression or death (PFS2) was an exploratory endpoint.

The study was registered at EudraCT (2007‐004823‐39) and ClinicalTrials.gov (NCT00689936). Written informed consent was provided by all patients. Institutional review boards or ethics committees at all sites approved the study before initiation. The study was conducted according to the Declaration of Helsinki and the Harmonization E6 Guidelines for Good Clinical Practice.

### 2.2 Treatment

In the Rd‐containing arms (28‐day cycles), oral lenalidomide (25 mg) was given on days 1 to 21 and oral dexamethasone (40 and 20 mg in patients ≤75 and >75 years of age, respectively) was given on days 1, 8, 15, and 22. Treatment was until disease progression (Rd continuous) or for 18 cycles (72 weeks, Rd18). In the MPT arm (42‐day cycles), oral melphalan (0.25 and 0.20 mg/kg in patients ≤75 and >75 years of age, respectively) was given on days 1 to 4, oral prednisone (2 mg/kg) was given on days 1 to 4, and oral thalidomide (200 and 100 mg in patients ≤75 and >75 years of age, respectively) was given daily. Treatment in the MPT arm was for 12 cycles (72 weeks). Details of starting dose adjustments for renal function and neutrophil counts were provided in a prior publication's supplementary appendix.^12^

### 2.3 Assessments

The data cutoff date (January 21, 2016) was the same used for the final analysis of OS.^12^ Response was assessed using the International Myeloma Working Group criteria for multiple myeloma.^18^ The National Cancer Institute Common Terminology Criteria for Adverse Events (version 3.0) was used to grade treatment‐emergent adverse events (TEAEs).^19^ Time‐to‐event endpoints (e.g., PFS and OS) were estimated using the Kaplan‐Meier product‐limit method. An unstratified log‐rank test was used to compare differences between treatment arms.

## 3 RESULTS

### 3.1 Patient characteristics

This subanalysis was conducted in the 312 patients from sites in Canada (n = 252) and the United States (n = 60), with 104 patients each in the Rd continuous, Rd18, and MPT arms (Table 1). The median age was 74 years, and 41.0% of patients were >75 years old. Baseline demographics were generally similar between the treatment arms, although the proportion of patients who were male was higher in the Rd continuous arm (61.5%) than the Rd18 and MPT arms (52.9% and 48.1%, respectively). Additionally, ECOG PS 0 frequency was lower in the MPT arm (17.3%) than the Rd‐containing arms (31.7% and 30.8% for Rd continuous and Rd18, respectively). Similarly, fewer patients had baseline creatinine clearance ≥60 mL/min in the MPT arm (41.3%) than the Rd‐containing arms (46.2% and 52.9%).

**TABLE 1:** Baseline demographics

	Rd Continuous	Rd18	MPT
	(n = 104)	(n = 104)	(n = 104)
Age, median (range), years	74 (49‐91)	73.5 (55‐84)	74 (58‐90)
>75 years, n (%)	43 (41.3)	41 (39.4)	44 (42.3)
Male, n (%)	64 (61.5)	55 (52.9)	50 (48.1)
ECOG PS, n (%)			
0	33 (31.7)	32 (30.8)	18 (17.3)
1	46 (44.2)	57 (54.8)	57 (54.8)
2	24 (23.1)	15 (14.4)	26 (25.0)
3	1 (1.0)	0	1 (1.0)
ISS stage, n (%)			
I or II	58 (55.8)	58 (55.8)	58 (55.8)
III	46 (44.2)	46 (44.2)	46 (44.2)
Lactate dehydrogenase, n (%)			
<200 U/L	79 (76.0)	80 (76.9)	77 (74.0)
≥200 U/L	25 (24.0)	24 (23.1)	27 (26.0)
Creatinine clearance, n (%)			
<30 ml/min	7 (6.7)	9 (8.7)	14 (13.5)
<60 ml/min	56 (53.8)	49 (47.1)	61 (58.7)
≥60 ml/min	48 (46.2)	55 (52.9)	43 (41.3)
History of bone lesions, n (%)	69 (66.3)	73 (70.2)	75 (72.1)
High‐risk cytogenetics, n (%)^a^	7 (6.7)	13 (12.5)	8 (7.7)

Abbreviations: ECOG PS, Eastern Cooperative Oncology Group performance status; ISS, International Staging System; MPT, melphalan +prednisone + thalidomide; Rd continuous, lenalidomide +dexamethasone until disease progression; Rd18, lenalidomide +dexamethasone for 18 cycles.

^a^ High‐risk cytogenetics included t(4;14), t(14;16), and del(17p).

### 3.2 Efficacy

**FIGURE 1:**
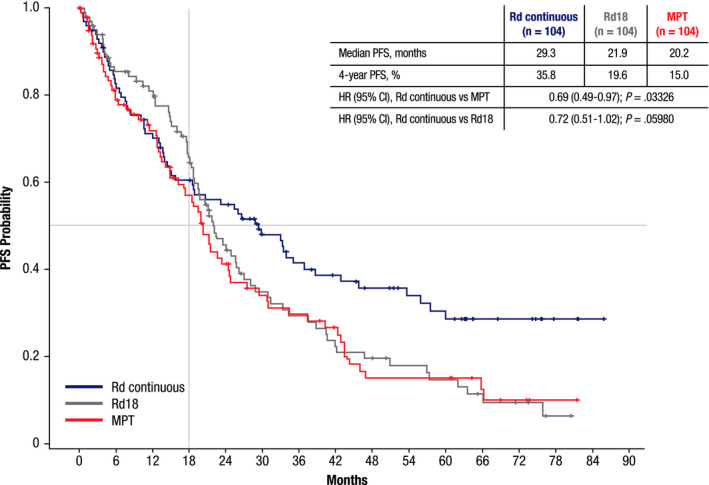
Progression‐free survival

Rd continuous demonstrated a significant improvement in PFS vs MPT (median, 29.3 vs 20.2 months; *p* = 0.03326; Figure 1) in the Canadian/US subgroup. Furthermore, the percentage of patients who were progression‐free at 4 years was more than doubled with Rd continuous vs MPT (35.8% vs 15.0%) and 1.8‐fold increased vs Rd18 (19.6%). The benefit of continuous vs fixed‐duration treatment was seen with a >7‐month improvement in median PFS with Rd continuous vs Rd18 (29.3 vs 21.9 months), although the difference did not reach statistical significance (*p* = 0.05980).

The benefit of Rd continuous was also seen in patients with deep responses. In patients achieving at least a very good partial response (VGPR), the median PFS was 56.0, 30.9, and 40.2 months with Rd continuous, Rd18, and MPT, respectively (Table 2).

**TABLE 2:** Efficacy

	Rd Continuous	Rd18	MPT
	(n = 104)	(n = 104)	(n = 104)
Overall response rate, n (%)	82 (78.8)	83 (79.8)	68 (65.4)
CR	22 (21.2)	23 (22.1)	12 (11.5)
VGPR	29 (27.9)	32 (30.8)	19 (18.3)
PR	31 (29.8)	28 (26.9)	37 (35.6)
≥VGPR	51 (49.0)	55 (52.9)	31 (29.8)
Time to next antimyeloma treatment			
Median, months	39.1	29.9	24.6
HR (95% CI), Rd continuous vs MPT	0.54 (0.37‐0.78); *p* = 0.00076		
HR (95% CI), Rd continuous vs Rd18	0.71 (0.49‐1.02); *p* = 0.06503		
PFS2			
Median, months	39.3	39.8	35.1
HR (95% CI), Rd continuous vs MPT	0.69 (0.50‐0.95); *p* = 0.02433		
Progression‐free survival in ≥VGPR			
Median, months	56.0	30.9	40.2
HR (95% CI), Rd continuous vs MPT	0.61 (0.34‐1.09)		
Overall survival in ≥VGPR			
Median, months	NR	80.1	NR
HR (95% CI), Rd continuous vs MPT	0.98 (0.49‐1.97)		

Abbreviations: CR, complete response; HR, hazard ratio; MPT, melphalan +prednisone + thalidomide; NR, not reached; PFS2, time from randomization to second progression or death; PR, partial response; Rd continuous, lenalidomide +dexamethasone until disease progression; Rd18, lenalidomide +dexamethasone for 18 cycles; VGPR, very good partial response.

**FIGURE 2:**
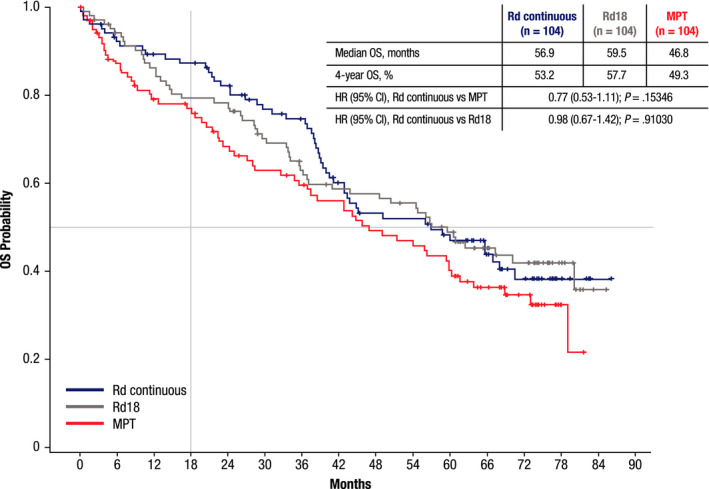
Overall survival

The median OS with Rd continuous vs MPT in the Canadian/US subgroup was 56.9 vs 46.8 months (*p* = 0.15346; Figure 2). The median OS with Rd18 was 59.5 months.

Rd continuous achieved a greater ORR than MPT (78.8% vs 65.4%). Furthermore, Rd continuous achieved deeper responses vs MPT, with ≥VGPR rates of 49.0% vs 29.8% and CR rates of 21.2% and 11.5%, respectively. In the Rd18 arm, the ORR was 79.8%, and 52.9% and 22.1% of patients achieved ≥VGPR and CR, respectively.

Median PFS2 was significantly longer with Rd continuous vs MPT (median, 39.3 vs 35.1 months; *p* = 0.02433) as was TTNT (median, 39.1 vs 24.6 months; *p* = 0.00076). Median PFS2 and TTNT with Rd18 were 39.8 and 29.9 months, respectively.

### 3.3 Safety

The median duration of treatment was 19.7 months (range, 0.5‐86.0 months) with Rd continuous, 16.6 months (range, 0.2‐19.5 months) with Rd18, and 14.6 months (range, 0.1‐23.5 months) with MPT. The mean duration of treatment, which takes into account the long‐term treatment of patients with Rd continuous, was 28.3, 13.1, and 11.7 months in the Rd continuous, Rd18, and MPT arms, respectively. The mean number of cycles was 28.9 (range, 1‐92) with Rd continuous, 13.6 (range, 1‐18) with Rd18, and 7.8 (range, 1‐12) with MPT.

In the Canadian/US subgroup, neutropenia was the most common grade 3/4 hematologic TEAE, with rates notably higher with MPT (52.0%) than with Rd continuous (28.4%) and Rd18 (30.1%; Table 3). Grade 3/4 anemia was also common, reported in 23.5%, 21.4%, and 23.5% of patients in the Rd continuous, Rd18, and MPT arms, respectively. Infections (grouped term) were the most common grade 3/4 nonhematologic TEAEs, reported in 37.3%, 30.1%, and 24.5% of patients in the Rd continuous, Rd18, and MPT arms, respectively. Analysis showed a higher rate of invasive hematologic SPMs with MPT (2.9%) vs Rd continuous (0%) and Rd18 (0%) (Table 4).

**TABLE 3:** Selected grade 3/4 treatment‐emergent adverse events

	Rd Continuous	Rd18	MPT
	(n = 102)	(n = 103)	(n = 102)
Hematologic, n (%)			
Neutropenia	29 (28.4)	31 (30.1)	53 (52.0)
Anemia	24 (23.5)	22 (21.4)	24 (23.5)
Thrombocytopenia	9 (8.8)	10 (9.7)	14 (13.7)
Febrile neutropenia	1 (1.0)	6 (5.8)	4 (3.9)
Nonhematologic, n (%)			
Infections	38 (37.3)	31 (30.1)	25 (24.5)
Pneumonia	11 (10.8)	14 (13.6)	9 (8.8)
Deep vein thrombosis	9 (8.8)	4 (3.9)	2 (2.0)
Cataract	7 (6.9)	4 (3.9)	0
Pulmonary embolism	5 (4.9)	2 (1.9)	2 (2.0)
Diarrhea	3 (2.9)	5 (4.9)	2 (2.0)
Peripheral sensory neuropathy	1 (1.0)	1 (1.0)	9 (8.8)
Constipation	0	2 (1.9)	3 (2.9)

Abbreviations: MPT, melphalan +prednisone + thalidomide; Rd continuous, lenalidomide +dexamethasone until disease progression; Rd18, lenalidomide +dexamethasone for 18 cycles.

**TABLE 4:** Second primary malignancies

	Rd Continuous	Rd18	MPT
	(n = 102)	(n = 103)	(n = 102)
Invasive, n (%)	7 (6.9)	8 (7.8)	12 (11.8)
Hematologic	0	0	3 (2.9)
MDS	0	0	2 (2.0)
MDS to AML	0	0	1 (1.0)
Solid tumor	7 (6.9)	8 (7.8)	9 (8.8)

Abbreviations: AML, acute myeloid leukemia; MDS, myelodysplastic syndromes; MPT, melphalan +prednisone + thalidomide; Rd continuous, lenalidomide +dexamethasone until disease progression; Rd18, lenalidomide +dexamethasone for 18 cycles.

## 4 DISCUSSION

The results of this Canadian/US subgroup are consistent with the findings from the ITT population of the phase 3 FIRST trial and confirm the benefit of Rd continuous over fixed‐duration Rd18 and MPT. Rd continuous extended median PFS by 9 months vs MPT and >7 months vs Rd18 in this subset of patients. Rd continuous also achieved deeper responses (≈20% increase in ≥VGPR rate) and delayed median TTNT by >14 months vs MPT. Furthermore, Rd continuous resulted in a longer PFS2 vs MPT, suggesting the benefit of frontline Rd continuous is maintained at relapse. Together, these results further support Rd continuous therapy as the standard of care for transplant‐ineligible patients with NDMM.

The demographic characteristics were generally similar between the treatment arms and between this subgroup and the ITT population.^11^ We do note, however, in the Canadian/US subgroup, the MPT arm had fewer patients with ECOG PS of 0 and creatinine clearance ≥60 ml/min compared with the Rd‐containing arms. Additionally, the MPT arm of the Canadian/US subgroup tended to be older (>75 years of age: 42% vs 34%) and had a less favorable performance status (ECOG PS of 0: 17% vs 29%) vs the ITT population. This may explain the differences in median PFS (20.2 vs 21.9 months) and OS (46.8 vs 49.1 months) in the MPT arm between the subgroup and ITT populations.^12^

Other phase 3 studies have confirmed the activity of Rd continuous in NDMM, including SWOG S0777 (in patients not intended for immediate transplant) and MAIA (in transplant‐ineligible patients).^20^, ^21^, ^22^ After a longer follow‐up of MAIA (Rd continuous ±daratumumab), median PFS in the Rd continuous arm was 33.8 months compared with 26.0 months in FIRST.^11^, ^12^, ^22^ This difference may reflect an increased knowledge and familiarity with Rd continuous, including an ability to maintain patients on therapy longer by addressing adverse events, from when the trials started (FIRST in 2008 and MAIA in 2015). The median duration of treatment was 18.4 months in FIRST vs 21.3 months at the time of primary endpoint analysis of the ongoing MAIA study.^12^, ^21^ This difference may impact conclusions drawn from cross‐trial comparisons with FIRST or other early Rd‐containing studies. For example, recent cross‐trial analyses of bortezomib +melphalan + prednisone (VMP) from the GIMEMA‐MM‐03‐05 trial vs Rd followed by lenalidomide maintenance from the EMN01 trial could be impacted by the median PFS of 18.6 months in the lenalidomide‐treated patients analyzed.^23^, ^24^

In contrast to the improvement in PFS with Rd continuous in subsequent trials, the median PFS in the VMP arms of the VISTA and ALCYONE trials were similar (18.3 and 19.3 months, respectively) despite a >10‐year gap between trials (enrollment initiated in 2004 and 2015, respectively).^25^, ^26^, ^27^, ^28^ Noting the limitations of cross‐trial comparisons, the higher PFS and improvement in outcomes in recent trials suggest that Rd continuous may be a better combination with which to add daratumumab than VMP. Indeed, the Rd‐based combination with daratumumab had a median PFS that was not yet reached, and 68% of patients were progression‐free at 36 months (after a median follow‐up of 36.4 months) vs the VMP‐based combination (median PFS, 36.4 months) in patients with transplant‐ineligible NDMM.^22^, ^28^

The safety results in the Canadian/US subgroup analysis were generally consistent with the profile in the ITT population, and no new safety concerns were observed. As noted for the ITT population, hematologic SPMs were less frequent in the Rd arms compared with the MPT arm, and incidence of solid tumor SPMs was similar across all treatment arms.

The improved outcomes with Rd continuous may derive from the immunomodulatory effects of lenalidomide. This hypothesis stems from results of studies in transplant‐eligible patients with NDMM, where differences in immune microenvironment after transplant are predictive of outcomes. For example, some (but not all) studies have shown that early lymphocyte recovery is predictive of improved survival outcomes following autologous stem cell transplant.^29^, ^30^, ^31^ Additionally, higher myeloma‐reactive T‐cell ratios of CD27^–^:CD27^+^ were prognostic for PFS, which may be reflective of reactivation after treatment with lenalidomide and subsequent transplant.^32^ The type of treatment may also impact immune recovery, as shown by a recent analysis of immune dysregulation (i.e., absolute lymphocyte and monocyte counts) within 1 month of novel‐agent treatment in a mixed (transplant‐eligible and ‐ineligible) NDMM population (N = 771).^33^ The highest rates of immune recovery and lowest rates of new immune dysregulation were in patients who received immunomodulatory agents alone vs those who received proteasome inhibitors alone or a combination of the two classes. Finally, lenalidomide has also been shown to augment systemic immunity and vaccine responses.^34^ Altogether, these results are hypothesis‐generating and need to be further explored in the transplant‐ineligible setting.

The post hoc nature of this analysis is a potential limitation. The smaller number of patients in the subanalysis also reduces the statistical power, which may explain why some outcomes that were significant in the ITT analysis did not reach significance in the subgroup population. The smaller population also limits the ability to conduct segmented comparisons (e.g., early vs late relapses, safety) and further compare outcomes based on baseline demographics, including age, cytogenetic risk, and renal function.

Overall, results of the Canadian/US subgroup analysis were consistent with those of the ITT population and support the role of Rd continuous as a standard of care for transplant‐ineligible patients with NDMM. Rd has been further investigated in combinations with bortezomib and daratumumab, resulting in improved outcomes and U.S. Food and Drug Administration approval.^20^, ^21^, ^22^, ^35^, ^36^ Trials investigating other Rd‐based combinations are also ongoing, including IMROZ (isatuximab, NCT03319667), EMN20 (carfilzomib, NCT04096066), and ELOQUENT 1 (elotuzumab, NCT01335399).

## CONFLICT OF INTEREST

AB, MCC, CS: nothing to disclose; NB: consultancy for and honoraria and research funding from Janssen, Bristol Myers Squibb Company, and Amgen; DW: honoraria from and board of directors or advisory committee participant for Amgen, Bristol Myers Squibb Company, Janssen, and Takeda; CC: honoraria from Bristol Myers Squibb Company; KS: honoraria and research funding from and board of directors or advisory committee participant for Bristol Myers Squibb Company; AT: consulting for Roche and Novartis; AD: research funding from Bristol Myers Squibb Company, Takeda, Alnylam, and Pfizer; advisory fees from Akcea, Janssen, Intellia, OncoTracker; KA: advisory board participant for Bristol Myers Squibb Company, Millennium, Takeda, Janssen, Gilead, and Sanofi; DB: employment with Bristol Myers Squibb Company; SR, SS: employment with and equity ownership in Celgene; TF: consulting/advisory fees from Amgen, Bristol Myers Squibb Company, Janssen, Karyopharm, PharmaMar, and Takeda; speakers bureau fees from Amgen, Bristol Myers Squibb Company, Janssen, and Takeda.

## Data Availability

Data requests may be submitted to Celgene, a Bristol Myers Squibb Company, at https://vivli.org/ourmember/celgene/ and must include a description of the research proposal.
